# Risk of Coronary Heart Disease in Different Criterion of Impaired Fasting Glucose: A Meta-Analysis

**DOI:** 10.1097/MD.0000000000001740

**Published:** 2015-10-09

**Authors:** Tianyu Xu, Wangkai Liu, Xiaoyan Cai, Jian Ding, Hongfeng Tang, Yuli Huang, Yunzhao Hu

**Affiliations:** From the Clinical Medicine Research Institute, The Affiliated Hospital at Shunde, Southern Medical University, Foshan (TX, XC, JD, HT, YH, YH); First School of Clinical Medicine, Southern Medical University (TX, JD); Department of Pediatrics, The First Affiliated Hospital, SUN Yat-sen University, Ghangzhou (WL); and Department of Cardiology, the First People's Hospital of Shunde, Foshan, P.R. China (YH).

## Abstract

The cut-point for diagnosing impaired fasting glucose (IFG) had been dispute, as reports about the associated clinical events are inconsistent. This meta-analysis evaluated the risk of coronary heart disease (CHD) in association with different criterion of IFG according to the American Diabetes Association (ADA) or the World Health Organization (WHO) Expert Group. We included prospective cohort studies with multivariate-adjusted data on IFG and CHD for analysis. The relative risks (RRs) of CHD were calculated and reported with 95% confidence intervals (95% CIs). Seventeen prospective cohort studies, comprising 527,021 individuals were included. The risks of CHD were increased in both participants with IFG defined as the ADA or WHO criterion (RR 1.11, 95% CI 1.02–1.21; and RR 1.18, 95% CI 1.10–1.28, respectively). Subgroup analyses showed that in both definition of IFG, the risk of CHD was only increased in studies with possibility of enrolling patients with increased 2 hours plasma glucose (2-h PG), or in studies with inadequate adjustment, but not in studies excluded participants with increased 2-h PG or in those with adequate adjustment of other risk factors. Our meta-analysis demonstrates that the presence of IFG was significantly associated with future risk of CHD. The risk of CHD was increased when fasting plasma glucose was as low as 100 mg/dL according to the lower cut-point of IFG by the ADA criterion. However, the risk maybe confounded by the undetected increased 2-h PG or other cardiovascular risk factors.

## Abstract 2

Supplemental Digital Content is available in the text

## INTRODUCTION

The term prediabetes is used to define individuals with intermediate states of abnormal dysglicemia between normoglycemia and overt type 2 diabetes mellitus (T2DM), including those with impaired fasting glucose (IFG) and those with impaired glucose tolerance (IGT).^1^ Subjects with IFG or IGT are at high risk for developing T2DM.^1^ It has also been reported that IGT is associated with increased risk of cardiovascular disease (CVD).^2,3^ However, the association of IFG and risk of CVD is far more unclear.^4^ Furthermore, the 2003 American Diabetes Association (ADA) guideline lowered the fasting plasma glucose (FPG) cut-point for diagnosing IFG from 110–125 to 100–125 mg/dL, in order to better identify subjects with future T2DM risk.^5^ Although more than a decade has passed, this change is still contentious and not adopted by the World Health Organization (WHO) Expert Group^6^ or other international guidelines.^7,8^ One of the main arguments against the cut-point of IFG proposed by 2003 ADA is that it greatly increases the number of subjects labeled with IFG, while without clear evidence of association with clinical complications.^9^ A recently published meta-analysis reported that the risk of stroke was increased in people with IFG defined as FPG 110 to 125 mg/dL (IFG 110) but not in those with IFG defined as FPG 100 to 125 mg/dL (IFG 100).^10^ However, another meta-analysis showed that the risks for CVD are similar in subjects with IFG 110 and IFG 100.^11^ These inconsistencies may be caused by the differences in inclusion criteria and endpoint assessment.

Considering these inconsistent results, we aimed to evaluate the association between different definitions of IFG and risk of coronary heart disease (CHD).

## METHODS

### Ethics Statement

This study does not involve patients, so ethical approval was not required.

### Search Strategy and Selection Criteria

The search strategy was performed in accordance with the recommendations of the Meta-analysis of Observational Studies in Epidemiology (MOOSE) Group.^12^ Electronic databases (PubMed and EMBASE) were searched for prospective cohort studies to May 31, 2015, using a combined text and MeSH heading search strategy with the terms “blood glucose,” “impaired fasting glucose,” “hyperglycaemia,” or “borderline diabetes” and “cardiovascular events,” “cardiovascular disease,” “ischemic heart disease,” “coronary heart disease,” “coronary artery disease,” “myocardial ischemia,” “myocardial infarction,” “angina” and “risk,” or “risk factors.” We restricted the search to human studies, but there were no language or publication form restrictions. The reference lists of published articles and reviews on this topic were also checked to identify other eligible studies. The detailed search strategy used for PubMed is presented in online supplementary Table S1, http://links.lww.com/MD/A453. The strategy for the EMBASE database was similar, but was adapted where necessary.

The inclusion criteria of studies for analysis were: prospective cohort studies involving adult participants (aged ≥18 years) with assessment of risk of CHD; blood glucose and other cardiovascular risk factors were evaluated at baseline; and adjusted relative risk (RR) and 95% confidence intervals (CIs) reported for events associated with IFG relative to normal fasting glucose (NFG). IFG defined as FPG of 100 to 125 mg/dL (IFG 100) or 110 to 125 mg/dL (IFG 110).^5,6^ Corresponding NFG comparator was defined as FPG < 100 or < 110 mg/dL, respectively.

Studies were excluded if: data were collected from patients with a particular condition (eg, previous history of hypertension, acute myocardial infarction, and kidney disease) but not general population; not accessed the risk of CHD in people with IFG compared with NFG; the risk of CHD in IFG was unadjusted for other risk factors; or reports were derived from the same cohort. If duplicate publications were identified as from the same cohort, only data from the most recent publication were used for analysis.^9,13^

### Data Extraction and Quality Assessment of Included Studies

Two authors (TY and WL) independently conducted independent literature searches, reviewed the potentially articles, and abstracted data from eligible studies. The quality assessment was evaluated according to the Newcastle–Ottawa Quality Assessment Scale (NOS) for assessing the quality of nonrandomized studies in meta-analyses,^14^ which based on the assessment of bias for selection, comparability, and exposure/outcome, with a total score up to 9. In this meta-analysis, included studies were graded as good quality if they with a score ≥7, fair if they had less than 7 score.^13^ We also evaluated whether the studies were adequate adjusted for potential confounders (at least 6 of 8 factors: age, sex, blood pressure or antihypertensive treatment, body mass index or other measure of overweight/obesity, physical activity, cholesterol concentration or lipid-lowering medication use, history of CVD or exclusion of CVD at baseline, and smoking).

### Data Synthesis and Analysis

We analyzed the RR of CHD in individuals with different definition of IFG. Subgroup analyses were conducted according to sex (women vs men), ethnicity (Asian vs non-Asian), specific end points (fatal vs fatal plus nonfatal CHD), participant's age (average <50 vs ≥50 years), follow-up duration (<10 vs ≥10 years), possibility of enrolling patients with diabetes (yes vs no), and adjustment of risk factors (adequate vs un-adequate).

We extracted the most adjusted RRs and 95% CIs from each included studies and logarithmically transformed these values, calculated the corresponding standard errors (SEs) to stabilize the variance and normalize the distribution.^15,16^ The inverse variance method was used to combine the log RRs and SEs using random effects models. The I^2^ statistic was used to estimate between-study heterogeneity. Values of I^2^ > 50% were considered to indicate significant heterogeneity. The estimated RRs were calculated using random-effects models. The test for subgroup differences was calculated by Chi-square statistics.

Publication bias was assessed by inspecting funnel plots for each outcome in which the natural log of RR was plotted against its SE. Sensitivity analyses were conducted by omitting one study at a time and recalculating the estimated RRs and CIs. *P* values were 2-tailed, and the statistical significance was set at 0.05. All analyses were performed with RevMan software (version 5.3 for Windows; The Cochrane Collaboration, Copenhagen, Denmark).

## RESULTS

### Studies Retrieved and Characteristics

**FIGURE 1:**
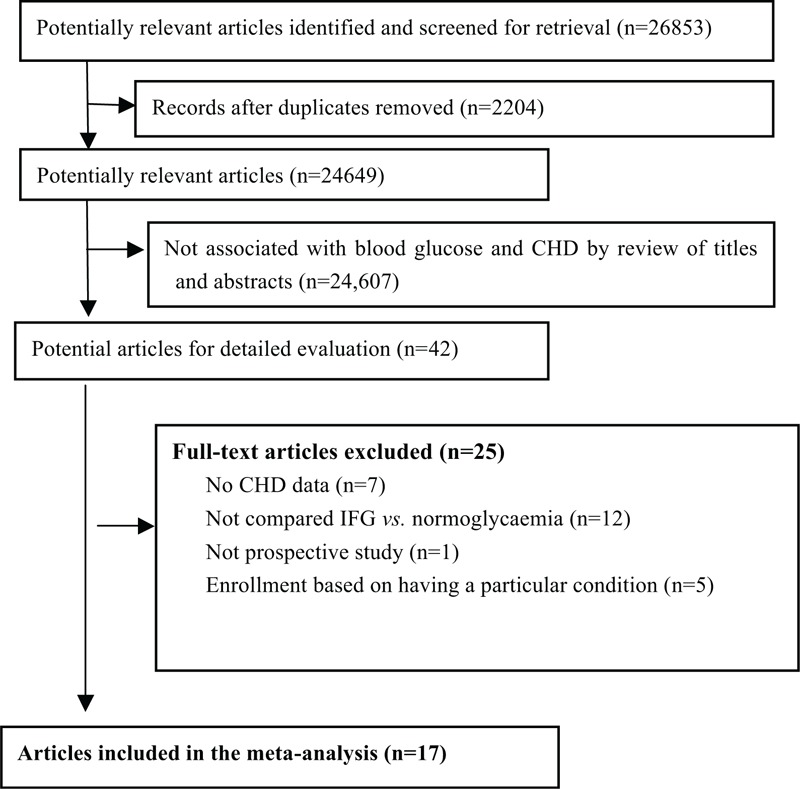
Flow of papers through review. CHD = coronary heart disease, CIs = confidence intervals, IFG = impaired fasting glucose, RR = relative risk.

A total of 26,853 manuscripts were retrieved in the initial search. After screening of the titles and abstracts, 42 reports qualified for full review. Finally, 17 prospective cohort studies, comprising 527,021 individuals, were included in our analysis^17–33^ (Fig. 1).

All of the included studies were derived from the general population. The characteristics of the 17 studies are presented in Table 1 . Nine of the studies were from the US and Europe^17,19–21,24–26,30,31^ and 8 were from Asia.^18,22,23,27–29,32,33^ One study only enrolled men^26^ while all of the others included both men and women for analysis. The follow-up duration ranged from 4 to 20 years.

**TABLE 1:**
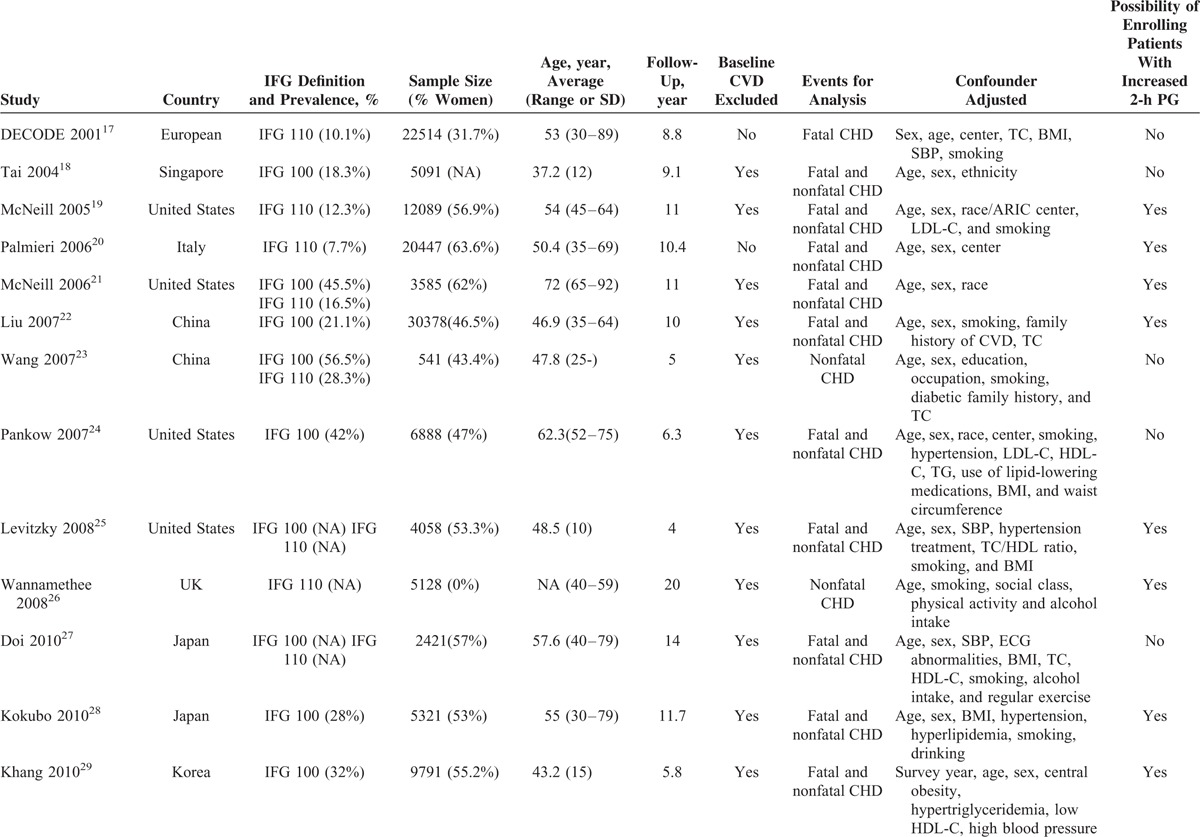
Study Characteristics

**TABLE 1 (Continued):**
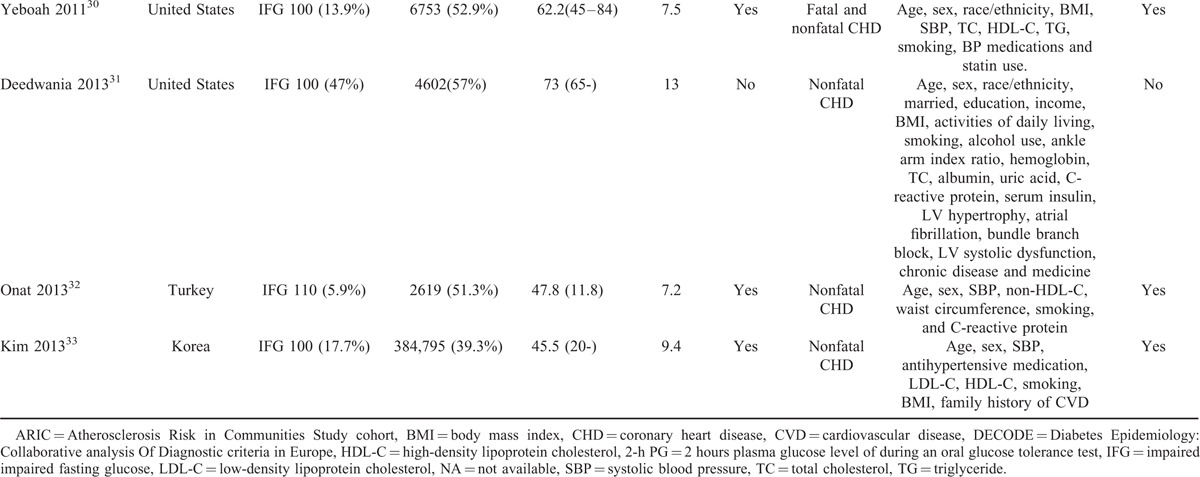
Study Characteristics

Oral glucose tolerance tests (OGTTs) were only performed in 6 studies and patients with increased 2 hours plasma glucose (2-h PG) were excluded for the analysis of risk in IFG.^17,18,23,24,27,31^ However, 11 studies only measured FPG at baseline without OGTT; therefore, these studies may enrolled patients with increased 2-h PG (IGT or T2DM defined by 2-h PG).^19–22,25,26,28–30,32,33^

All studies were graded as good quality accessed by the NOS. The details of the quality assessment are presented in Supplemental Table 2, http://links.lww.com/MD/A453. Furthermore, according to the confounders adjusted, 7 studies did not meet our criteria for adequate adjustment^18–23,26^ and 10 studies were adequate adjusted for other potential confounders.^17,24,25,27–33^

### Association Between IFG and Risk of CHD

**FIGURE 2:**
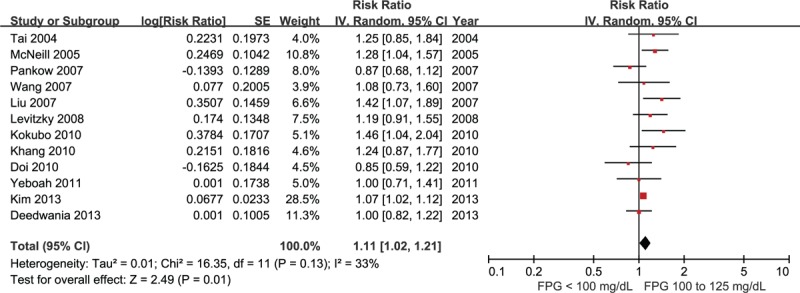
Forest plot of the comparison: IFG 100 versus normoglycemia, outcome: coronary heart disease. IFG 100 = impaired fasting glucose (fasting glucose 100–125 mg/dL).

Twelve studies comprising 475,347 participants reported data for risk of CHD associated with IFG 100 compared with NFG, defined as FPG <100 mg/dL.^18,19,22–25,27–31,33^ There was moderate between-study heterogeneity in these studies (I^2^ = 33%). Meta-analysis using random-effects models showed that the risk of CHD was significantly increased in individuals with IFG 100 (RR 1.11, 95% CI 1.02–1.21, Fig. 2).

**FIGURE 3:**
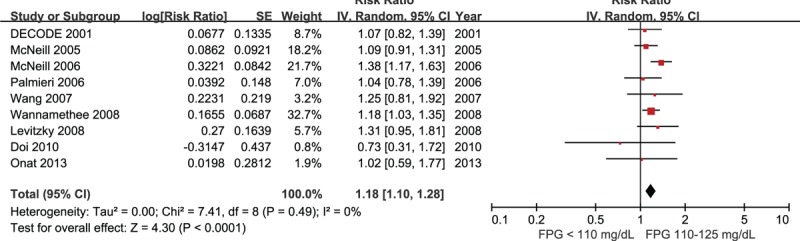
Forest plot of the comparison: IFG 110 versus normoglycemia, outcome: coronary heart disease. IFG 110 = impaired fasting glucose (fasting glucose 110–125 mg/dL).

Nine studies comprising 73,402 participants were included for the analysis of risk of CHD in IFG 110 compared with FPG <110 mg/dL.^17,19–21,23,25–27,32^ There was no between-study heterogeneity detected in these studies (I^2^ = 0%), and the risk of CHD was significantly increased in individuals with IFG 110 (RR 1.18, 95% CI 1.10–1.28, Fig. 3).

Visual inspection of funnel plots suggested that there was no evidence of publication bias for either IFG 100 (supplementary Figure S1, http://links.lww.com/MD/A453) or IFG 110 group (supplementary Figure S2, http://links.lww.com/MD/A453).

Sensitivity analyses confirmed that the risk of CHD in people with IFG 100 or IFG 110 were not influenced by the use of random-effects models compared with fixed-effects models, or recalculating the RRs by omitting one study at a time.

### Subgroup Analyses

The results of subgroup analyses are presented in Table 2. In individuals with IFG 100, there were no significantly differences among subgroups conducted according to sex, ethnicity, participant's age, specific end points, and follow-up duration. However, the risk of CHD was significantly increased in studies with possibility of enrolling patients with increased 2-h PG (RR 1.19, 95% CI 1.06–1.33), but not in studies excluded participants with increased 2-h PG (RR 0.98, 95% CI 0.86–1.11). Furthermore, the risk of CHD was increased in studies with inadequate adjustment (RR 1.27, 95% CI 1.12–1.45), but not in those with adequate adjustment of other risk factors (RR 1.05, 95% CI 0.96–1.15). The differences of CHD risk between these subgroups comparison were both significant (both *P* = 0.02).

**TABLE 2:**
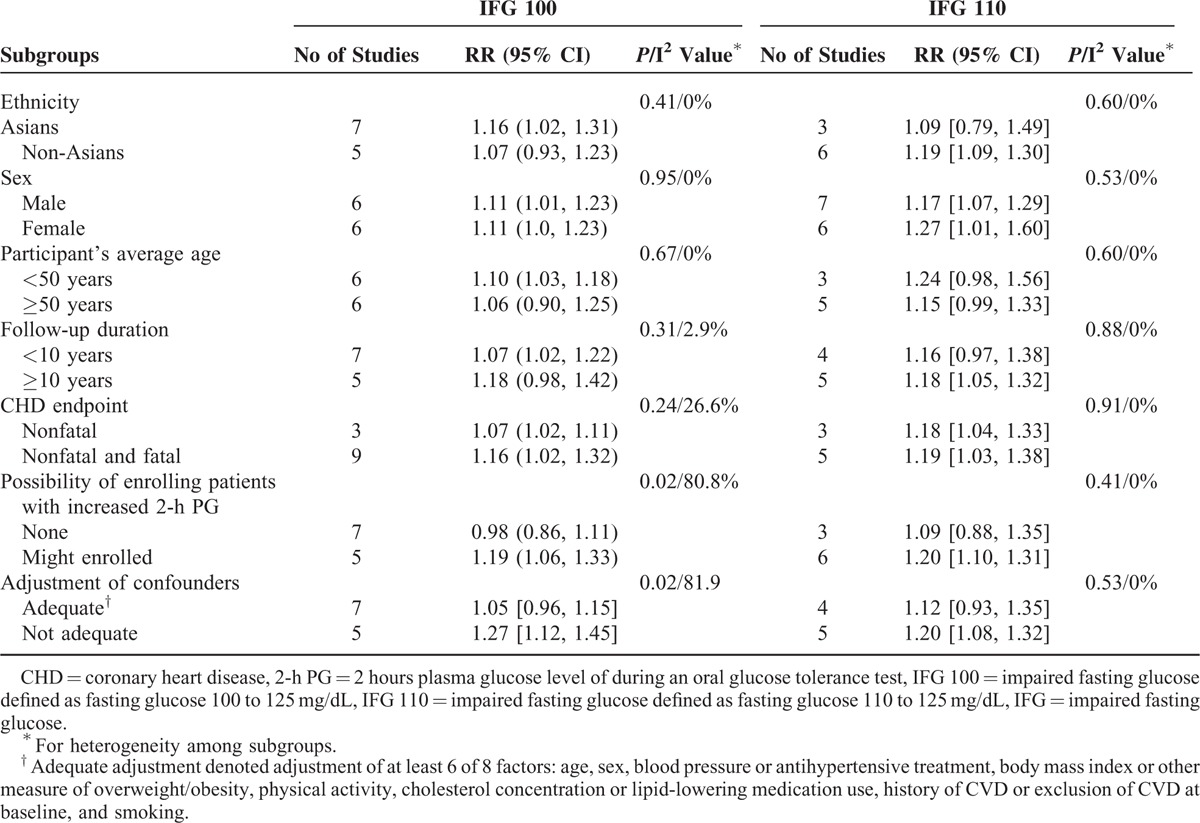
Subgroup Analyses of the Association Between IFG and Risk of CHD

In subgroups analysis of IFG 110, the risk of CHD was also increased in studies with possibility of enrolling patients with increased 2-h PG (RR 1.20, 95% CI 1.10–1.31), not in studies excluded participants with increased 2-h PG (RR 1.09, 95% CI 0.88–1.35), in studies with inadequate adjustment (RR 1.20, 95% CI 1.08–1.32), but not in those with adequate adjustment of other risk factors (RR 1.12, 95% CI 0.93–1.35). However, there were no significant differences among all subgroups comparison (all *P* > 0.1, I^2^ = 0%).

## DISCUSSION

In this meta-analysis, we found that in the general population, IFG was significantly associated with future risk of CHD. The risk of CHD was increased when FPG was as low as 100 mg/dL according to the lower cut-point of IFG by the ADA.

The 2003 ADA criterion of IFG had been criticized as it significantly increased the prevalence of IFG while without improvement of prediction for risk of CVD.^34^ In this study, there was sufficient power to show that the presence of IFG, defined by the WHO or ADA criterion, was associated with increased risk of CHD. These findings support the lower IFG cut-point proposed by the ADA and highlight the importance of early management of mild hyperglycemia for the prevention of CHD. Our results were different with a prior meta-analysis, which showed that the risk of stroke was increased in people with IFG defined by the WHO but not in those defined by the ADA.^10^ These inconsistent findings may be caused by differences in the events assessed. Furthermore, in the prior meta-analysis, they combined studies from general population, as well as studies from patients with coronary artery disease for analysis.^10^ However, we only used studies from general population for analysis. Our more stringent inclusion criteria are important for avoiding between-study heterogeneity and reaching more reliable conclusion. In our study, the risk of CHD associated with IFG was significantly increased in studies with possibility of enrolling patients with increased 2-h PG, but not in studies excluded participants with increased 2-h PG. These results showed that the risk of CHD in people with FPG maybe confounded by the undetected increased 2-h PG (IGT or T2DM defined by 2-h PG). Many studies have shown that IGT was a stronger predictor of cardiovascular events than IFG.^17,35^ However, routine detection of IGT had been questioned due to the inconvenient use of OGTT and the results are not highly reproducible. Our results highlight the notion that OGTT could be required for further diagnosing individuals with IFG.^36^

It has been estimated that, by the year of 2025, the number of people with prediabetes will be 472 millions.^37^ Successful interventions in this large population could have a major public health impact. It had been proved that lifestyle is a fundamental management approach that can effectively prevent the progression from prediabetes to diabetes.^38^ Furthermore, recently data showed that lifestyle intervention in IGT can reduce incidence of cardiovascular and all-cause mortality.^39^ However, the evidence regarding CVD prevention in people with IFG is still absent.

The main strengths of our study are the very large sample size with general population included from prospective cohort studies. Detailed subgroup analyses also found interesting results that the risk of CHD associated with FPG may be confounded by the undetected increased 2-h PG and other cardiovascular risk factors. However, our study also has some limitations. First, individuals with IFG are more likely to progress to DM than those with normoglycemia,^1^ but most of the included studies did not adjust for subsequent blood glucose levels. So, the long-term risk of CHD in people with IFG was caused by the mild elevation of blood glucose or the future progression of DM remains unknown. However, it had been indicated that coronary atherosclerosis detected by intravascular imaging modalities is already ongoing in prediabetic status.^40^ Second, the adjusted confounders in the included studies were inconsistent and may be a potential source of bias in our study. However, it is interesting that, in both IFG 100 and IFG 110 subgroup analysis, the risk of CHD was increased in studies with inadequate adjustment, but not in those with adequate adjustment of other cardiovascular risk factors. These results reinforce the importance of detection of other cardiovascular risk factors in risk stratification of people with IFG.^41^

In conclusion, this meta-analysis showed that IFG was associated with an increased risk of CHD. The risk increased in people with FPG as low as 100 mg/dL. These results reaffirm the importance of screening for prediabetes using the ADA criteria. Furthermore, detection of 2-h PG and other cardiovascular risk factors are important for risk stratification in people with IFG. These informations are important for the prevention of DM and CVD.

## References

[1] Ferrannini E Definition of intervention points in prediabetes. *Lancet Diabetes Endocrinol* 2014; 2:667–675. 24731662 10.1016/S2213-8587(13)70175-X

[2] Buysschaert M Medina JL Bergman M Prediabetes and associated disorders. *Endocrine* 2015; 48:371–393. 25294012 10.1007/s12020-014-0436-2

[3] Faerch K Vistisen D Johansen NB Cardiovascular risk stratification and management in pre-diabetes. *Curr Diab Rep* 2014; 14:493. 24743942 10.1007/s11892-014-0493-1

[4] De Caterina R Madonna R Impaired fasting plasma glucose and long-term cardiovascular risk: still a foggy relationship. *Eur Heart J* 2010; 31:1159–1162. 20418342 10.1093/eurheartj/ehp589

[5] Expert Committee on the Diagnosis and Classification of Diabetes Mellitus Report of the expert committee on the diagnosis and classification of diabetes mellitus. *Diabetes Care* 2003; 26 (Suppl 1):S5–S20. 12502614 10.2337/diacare.26.2007.s5

[6] World Health Organization (WHO) Consultation. Definition and diagnosis of diabetes and intermediate hyperglycaemia. 2006 http://www.who.int/diabetes/publications/Definition%20and%20diagnosis%20of%20diabetes_new.pdf [Accessed December 30, 2004]

[7] Ryden L Grant PJ Anker SD ESC Guidelines on diabetes, pre-diabetes, and cardiovascular diseases developed in collaboration with the EASD: the task force on diabetes, pre-diabetes, and cardiovascular diseases of the European Society of Cardiology (ESC) and developed in collaboration with the European Association for the Study of Diabetes (EASD). *Eur Heart J* 2013; 34:3035–3087. 23996285 10.1093/eurheartj/eht108

[8] Forouhi NG Balkau B Borch-Johnsen K The threshold for diagnosing impaired fasting glucose: a position statement by the European Diabetes Epidemiology Group. *Diabetologia* 2006; 49:822–827. 16525842 10.1007/s00125-006-0189-4

[9] Huang Y Cai X Chen P Associations of prediabetes with all-cause and cardiovascular mortality: a meta-analysis. *Ann Med* 2014; 46:684–692. 25230915 10.3109/07853890.2014.955051

[10] Lee M Saver JL Hong KS Effect of pre-diabetes on future risk of stroke: meta-analysis. *BMJ* 2012; 344:e3564. 22677795 10.1136/bmj.e3564 PMC3370083

[11] Ford ES Zhao G Li C Pre-diabetes and the risk for cardiovascular disease: a systematic review of the evidence. *J Am Coll Cardiol* 2010; 55:1310–1317. 20338491 10.1016/j.jacc.2009.10.060

[12] Stroup DF Berlin JA Morton SC Meta-analysis of observational studies in epidemiology: a proposal for reporting. Meta-analysis of Observational Studies in Epidemiology (MOOSE) group. *JAMA* 2000; 283:2008–2012. 10789670 10.1001/jama.283.15.2008

[13] Huang Y Cai X Qiu M Prediabetes and the risk of cancer: a meta-analysis. *Diabetologia* 2014; 57:2261–2269. 25208757 10.1007/s00125-014-3361-2

[14] GA Wells B Shea D O’Connell The Newcastle-Ottawa Scale (NOS) for assessing the quality of nonrandomised studies in meta-analyses. [serial online]. http://www. ohri.ca/programs/clinical_epidemiology/oxford.asp [Accessed January 1, 2008].

[15] Huang Y Cai X Liu C Prehypertension and the risk of coronary heart disease in Asian and Western populations: a meta-analysis. *J Am Heart Assoc* 2015; 4:pii: e001519. 10.1161/JAHA.114.001519 PMC4345875 25699996

[16] Huang Y Wang S Cai X Prehypertension and incidence of cardiovascular disease: a meta-analysis. *BMC Med* 2013; 11:177. 23915102 10.1186/1741-7015-11-177 PMC3750349

[17] DECODE Study Group, the European Diabetes Epidemiology Group Glucose tolerance and cardiovascular mortality: comparison of fasting and 2-hour diagnostic criteria. *Arch Intern Med* 2001; 161:397–405. 11176766 10.1001/archinte.161.3.397

[18] Tai ES Goh SY Lee JJ Lowering the criterion for impaired fasting glucose: impact on disease prevalence and associated risk of diabetes and ischemic heart disease. *Diabetes Care* 2004; 27:1728–1734. 15220254 10.2337/diacare.27.7.1728

[19] McNeill AM Rosamond WD Girman CJ The metabolic syndrome and 11-year risk of incident cardiovascular disease in the atherosclerosis risk in communities study. *Diabetes Care* 2005; 28:385–390. 15677797 10.2337/diacare.28.2.385

[20] Palmieri L Donfrancesco C Giampaoli S Favorable cardiovascular risk profile and 10-year coronary heart disease incidence in women and men: results from the Progetto CUORE. *Eur J Cardiovasc Prev Rehabil* 2006; 13:562–570. 16874146 10.1097/01.hjr.0000221866.27039.4b

[21] McNeill AM Katz R Girman CJ Metabolic syndrome and cardiovascular disease in older people: the cardiovascular health study. *J Am Geriatr Soc* 2006; 54:1317–1324. 16970637 10.1111/j.1532-5415.2006.00862.x

[22] Liu J Grundy SM Wang W Ten-year risk of cardiovascular incidence related to diabetes, prediabetes, and the metabolic syndrome. *Am Heart J* 2007; 153:552–558. 17383293 10.1016/j.ahj.2007.01.003

[23] Wang JJ Li HB Kinnunen L How well does the metabolic syndrome defined by five definitions predict incident diabetes and incident coronary heart disease in a Chinese population? *Atherosclerosis* 2007; 192:161–168. 16720024 10.1016/j.atherosclerosis.2006.04.027

[24] Pankow JS Kwan DK Duncan BB Cardiometabolic risk in impaired fasting glucose and impaired glucose tolerance: the Atherosclerosis Risk in Communities Study. *Diabetes Care* 2007; 30:325–331. 17259502 10.2337/dc06-1457

[25] Levitzky YS Pencina MJ D’Agostino RB Impact of impaired fasting glucose on cardiovascular disease: the Framingham Heart Study. *J Am Coll Cardiol* 2008; 51:264–270. 18206734 10.1016/j.jacc.2007.09.038

[26] Wannamethee SG The metabolic syndrome and cardiovascular risk in the British Regional Heart Study. *Int J Obes (Lond)* 2008; 32 (Suppl 2):S25–S29. 18469839 10.1038/ijo.2008.32

[27] Doi Y Ninomiya T Hata J Impact of glucose tolerance status on development of ischemic stroke and coronary heart disease in a general Japanese population: the Hisayama study. *Stroke* 2010; 41:203–209. 19940278 10.1161/STROKEAHA.109.564708

[28] Kokubo Y Okamura T Watanabe M The combined impact of blood pressure category and glucose abnormality on the incidence of cardiovascular diseases in a Japanese urban cohort: the Suita Study. *Hypertens Res* 2010; 33:1238–1243. 20927111 10.1038/hr.2010.174

[29] Khang YH Cho SI Kim HR Risks for cardiovascular disease, stroke, ischaemic heart disease, and diabetes mellitus associated with the metabolic syndrome using the new harmonised definition: findings from nationally representative longitudinal data from an Asian population. *Atherosclerosis* 2010; 213:579–585. 20940070 10.1016/j.atherosclerosis.2010.09.009

[30] Yeboah J Bertoni AG Herrington DM Impaired fasting glucose and the risk of incident diabetes mellitus and cardiovascular events in an adult population: MESA (Multi-Ethnic Study of Atherosclerosis). *J Am Coll Cardiol* 2011; 58:140–146. 21718910 10.1016/j.jacc.2011.03.025 PMC3146297

[31] Deedwania P Patel K Fonarow GC Prediabetes is not an independent risk factor for incident heart failure, other cardiovascular events or mortality in older adults: findings from a population-based cohort study. *Int J Cardiol* 2013; 168:3616–3622. 23731526 10.1016/j.ijcard.2013.05.038 PMC3939803

[32] Onat A Can G Cicek G Fasting, non-fasting glucose and HDL dysfunction in risk of pre-diabetes, diabetes, and coronary disease in non-diabetic adults. *Acta Diabetol* 2013; 50:519–528. 21769500 10.1007/s00592-011-0313-x

[33] Kim HK Kim CH Kim EH Impaired fasting glucose and risk of cardiovascular disease in Korean men and women: the Korean Heart Study. *Diabetes Care* 2013; 36:328–335. 23002083 10.2337/dc12-0587 PMC3554281

[34] Kim SH Chunawala L Linde R Comparison of the 1997 and 2003 American Diabetes Association classification of impaired fasting glucose: impact on prevalence of impaired fasting glucose, coronary heart disease risk factors, and coronary heart disease in a community-based medical practice. *J Am Coll Cardiol* 2006; 48:293–297. 16843178 10.1016/j.jacc.2006.03.043

[35] Wang J Ruotsalainen S Moilanen L The metabolic syndrome predicts cardiovascular mortality: a 13-year follow-up study in elderly non-diabetic Finns. *Eur Heart J* 2007; 28:857–864. 17303589 10.1093/eurheartj/ehl524

[36] Buysschaert M Bergman M Definition of prediabetes. *Med Clin North Am* 2011; 95:289–297.vii. 21281833 10.1016/j.mcna.2010.11.002

[37] Wild S Roglic G Green A Global prevalence of diabetes: estimates for the year 2000 and projections for 2030. *Diabetes Care* 2004; 27:1047–1053. 15111519 10.2337/diacare.27.5.1047

[38] Tuomilehto J Lindstrom J Eriksson JG Prevention of type 2 diabetes mellitus by changes in lifestyle among subjects with impaired glucose tolerance. *N Engl J Med* 2001; 344:1343–1350. 11333990 10.1056/NEJM200105033441801

[39] Li G Zhang P Wang J Cardiovascular mortality, all-cause mortality, and diabetes incidence after lifestyle intervention for people with impaired glucose tolerance in the Da Qing Diabetes Prevention Study: a 23-year follow-up study. *Lancet Diabetes Endocrinol* 2014; 2:474–480. 24731674 10.1016/S2213-8587(14)70057-9

[40] Kurihara O Takano M Seino Y Coronary atherosclerosis is already ongoing in pre-diabetic status: Insight from intravascular imaging modalities. *World J Diabetes* 2015; 6:184–191. 25685289 10.4239/wjd.v6.i1.184 PMC4317311

[41] Qiu M Shen W Song X Effects of prediabetes mellitus alone or plus hypertension on subsequent occurrence of cardiovascular disease and diabetes mellitus: longitudinal study. *Hypertension* 2015; 65:525–530. 25624343 10.1161/HYPERTENSIONAHA.114.04632

